# Obtaining Ready-to-Eat Blue Corn Expanded Snacks with Anthocyanins Using an Extrusion Process and Response Surface Methodology

**DOI:** 10.3390/molecules191221066

**Published:** 2014-12-15

**Authors:** Anayansi Escalante-Aburto, Benjamín Ramírez-Wong, Patricia Isabel Torres-Chávez, Jaime López-Cervantes, Juan de Dios Figueroa-Cárdenas, Jesús Manuel Barrón-Hoyos, Ignacio Morales-Rosas, Néstor Ponce-García, Roberto Gutiérrez-Dorado

**Affiliations:** 1 Programa de Doctorado en Ciencias de los Alimentos, Universidad de Sonora, Blvd. Luis Encinas y Rosales s/n, Hermosillo, Sonora 83000, Mexico; E-Mails: lic_anayansi@hotmail.com (A.E.-A.); pitorres@guayacan.uson.mx (P.I.T.-C.); jbarron@guaymas.uson.mx (J.M.B.-H.); imorales@guayacan.uson.mx (I.M.-R.); 2 Centro de Investigación e Innovación en Biotecnología Agropecuaria, Instituto Tecnológico de Sonora, 5 de Febrero 818 Sur, Col. Centro, Ciudad Obregón, Sonora 8500, Mexico; E-Mail: jaimelopez@bioderpac.com; 3 Centro de Investigación y Estudios Avanzados (CINVESTAV—Unidad Querétaro), Libramiento Norponiente#2000, Fraccionamiento Real de Juriquilla, Querétaro, Querétaro 76230, Mexico; E-Mail: jfigueroa@qro.cinvestav.mx; 4 UAEMex Campus Universitario “El Cerrillo”. El Cerrillo Piedras Blancas s/n, Toluca, Estado de Mexico 50200, Mexico; E-Mail: nponceg@uaemex.mx; 5 Programa Regional del Noroeste para el Doctorado en Biotecnología, Universidad Autónoma de Sinaloa, Av. de las Américas y Blvd. Universitarios s/n, Culiacán, Sinaloa 80010, Mexico; E-Mail: robe399@hotmail.com

**Keywords:** blue corn, anthocyanins, snack, extrusion, optimization

## Abstract

Extrusion is an alternative technology for the production of nixtamalized products. The aim of this study was to obtain an expanded nixtamalized snack with whole blue corn and using the extrusion process, to preserve the highest possible total anthocyanin content, intense blue/purple coloration (color *b*) and the highest expansion index. A central composite experimental design was used. The extrusion process factors were: feed moisture (FM, 15%–23%), calcium hydroxide concentration (CHC, 0%–0.25%) and final extruder temperature (T, 110–150 °C). The chemical and physical properties evaluated in the extrudates were moisture content (MC, %), total anthocyanins (TA, mg·kg^−1^), pH, color (*L*, *a*, *b*) and expansion index (EI). ANOVA and surface response methodology were applied to evaluate the effects of the extrusion factors. FM and T significantly affected the response variables. An optimization step was performed by overlaying three contour plots to predict the best combination region. The extrudates were obtained under the following optimum factors: FM (%) = 16.94, CHC (%) = 0.095 and T (°C) = 141.89. The predicted extrusion processing factors were highly accurate, yielding an expanded nixtamalized snack with 158.87 mg·kg^−1^ TA (estimated: 160 mg·kg^−1^), an EI of 3.19 (estimated: 2.66), and color parameter *b* of −0.44 (estimated: 0.10).

## 1. Introduction

The energy density of ingested foods has increased globally; populations have become more urban, and the high consumption of products with large amounts of sugar, carbohydrates, dyes and saturated fats has been reported [1]. There is also an increase in the worldwide consumption of cereal-based foods, especially snack products. This market is expanding rapidly and will continue growing in the coming years. Snack foods are considered high energy density products, and they are directly related to promoting weight gain and to causing certain illnesses such as obesity and other related diseases (metabolic syndrome, cardiovascular events, hypertension, cancer) [1].

Nixtamalized corn snacks are consumed mostly by the American and Latin-American population. On the other hand, the extrusion process (EP) has been used as a rapid and efficient technology to obtain a large variety of products, including nixtamalized products. The EP has some advantages compared to the traditional nixtamalization process, including requiring less time and energy input and no production of water effluents (nejayote).

Producing extruded snacks with whole grains, such as corn, improves the nutritional quality of the final product. The germ of the grain is composed of polyunsaturated acids, the pericarp contains dietary fiber, and phytochemicals (bioactive ingredients) such as anthocyanins are present in the outer part of the endosperm (aleurone layer). These compounds have been isolated from pigmented corn grains and have shown health benefits such as anti-radical activity, which plays an important role in preventing chronic-degenerative illnesses [2].

The chemical structure of anthocyanins is based on the aglycon molecule (the flavylium ion or 2-phenylbenzopirilium) with various substitutions of methoxyl and hydroxyl groups in different positions. Generally, these compounds are linked to one or more glycosidic molecules (hexoses and pentoses) and they also can be joined to different organic acids (cinnamic and aliphatic acids). Nevertheless, anthocyanins are unstable compounds at high pH levels and temperatures [3].

Anthocyanins are soluble compounds responsible for the blue/purple coloration in pigmented corns. As the chemical forms of anhocyanins change, their color also changes. The modification of certain process conditions such as pH and temperature, can produce the formation of a bluish quinoidal base or a colorless carbinol pseudobase [4]. These aspects affect the color of the products made with pigmented corns, modifying their appearance and acceptance.

Several investigations have focused on products obtained from pigmented corns produced by traditional, ecological and extrusion nixtamalization processes [5,6,7,8,9,10]. Nixtamalization by extrusion diminishes the anthocyanin losses in the end products by up to 50%–60% (tortillas and expanded extrudates), and ecological nixtamalization reduced the pH levels and the formation of compounds with proteins and carbohydrates that positively affected the retention of nutraceutical compounds [11]. All of those investigations are focused in improving the anthocyanins retention, in order to develop a healthy product with acceptable sensorial characteristics, which is the goal of food technologists.

It has been established that there are advantages to using whole grains with additional nutraceutical value (phytochemicals) to diminish the prevalence of chronic degenerative diseases. Innovations in snack products that already are on the market, lead to finding new ways of processing that result in improved products with low amounts of carbohydrates, dyes, saturated fats and low energy density.

The aim of this study was to produce expanded nixtamalized expanded snacks using extrusion process with whole blue corn, and to apply response surface methodology to obtain a product with a high total anthocyanin content, an intense purple/blue coloration, and a high expansion index.

## 2. Results and Discussion

### 2.1. Effects of FM, CHC and T on Extrudates Moisture Content (MC)

The second order equations coefficients, analysis of variance (ANOVA) and determination coefficients of feed moisture, final extruder temperature and calcium hydroxide concentration effect on the chemical and physical properties of the extrudates, are presented in Table 1.

The ANOVA showed that the FM was the processing variable that most affected the MC in linear terms (*p* < 0.0001). The quadratic terms (FM)^2^ and (T)^2^ presented a significant and a very significant effect on MC (*p* < 0.0152 and *p* < 0.0001, respectively).

The model fitting for MC in terms of the actual factors is presented in Equation (1):

Y_MC_ = −160.23 + 3.57(FM) + 2.07(T) + 0.009 (FM)(T) − 0.11(FM)^2^ − 0.008(T)^2^ (1)

**Figure 1:**
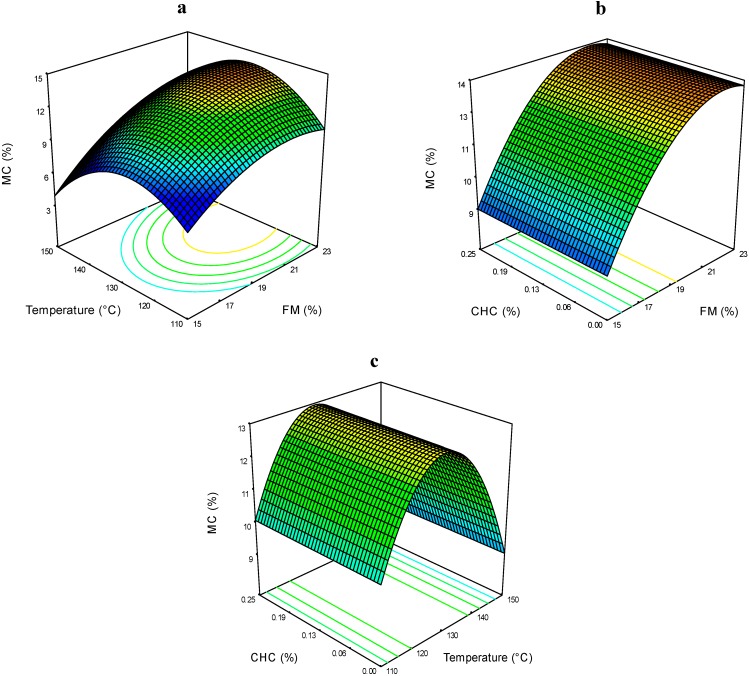
Moisture content (MC) of expanded nixtamalized blue corn extrudates, as a function of: (**a**) feed moisture (FM) and temperature (T); (**b**) feed moisture (FM) and calcium hydroxide concentration (CHC); and (**c**) temperature (T) and calcium hydroxide concentration (CHC).

The extrudates MC varied from 8.1% to 14.7% (Table 2). Figure 1a shows that higher values of FM yielded the highest MC in the extrudates at a T of 130 °C. In Figure 1b, the interaction of FM*CHC shows that at low FM content, the CHC had no effects the on extrudate MC. The interaction effect of T*CHC is presented in Figure 1c, where T showed an interesting effect; at 130 °C, the extrudates reached the highest MC regarding the CHC level.

**Table 1:** Coefficients of the second order equations (prediction models), analysis of variance and determination coefficients, showing the relationship among the processing factors and the chemical and physical properties of the extrudates.

Coefficients	MC ^a^	TA	pH	*L*	*a*	*b*	EI
**Intercept**							
β	13.45	147.63	6.85	21.61	3.87	−0.27	2.13
**Lineal**							
β_1_	1.51 ***	5.55ns	0.021ns	−2.54 ***	−0.35 ***	−0.23 ***	−0.45 ***
β_2_	−0.33ns	1.93ns	−0.0009ns	0.46ns	0.18 ***	−0.016ns	0.002ns
β_3_	0.050ns	0.39ns	−0.013ns	0.29ns	0.011ns	−0.004ns	−0.015ns
**Quadratic**							
β_11_	−0.66 **	2.86ns	−0.008ns	1.11 ***	0.17 ***	0.13 **	0.11 **
β_22_	−1.29 ***	7.39 *	−0.035ns	0.31ns	0.23 ***	0.030ns	−0.009ns
β_33_	−0.40ns	2.05ns	−0.001ns	0.38ns	−0.0007ns	0.063ns	0.001ns
**Interaction**							
β_12_	0.27ns	−4.94ns	−0.006ns	−0.62ns	−0.15 **	−0.005ns	−0.005ns
β_13_	0.25ns	3.26ns	0.0003ns	−0.016ns	−0.16 **	0.020ns	−0.035ns
β_23_	−0.005ns	−1.66ns	0.020ns	0.53ns	0.033ns	0.010ns	0.021ns
R^2^	0.89	0.44	0.17	0.89	0.92	0.72	0.95

^a^ MC = moisture content; TA = total anthocyanins; *L* = white (100) to black (0), *a* = red (+) to green (−), *b* = yellow (+) to blue (−), EI = Expansion index; β_1_, feed moisture; β_2_, final extruder temperature; β_3_, calcium hydroxide concentration. ns = not significant (*p* > 0.1); * *p* < 0.1; ** Significant (*p* < 0.05); *** Very significant (*p* < 0.01).

**Table 2:** Experiment design used to obtain different combinations of extrusion feed moisture/calcium hydroxide concentration/temperature for production of expanded nixtamalized blue corn extrudates.

Process Factors ^a^					Response Variables ^b^					
Tr ^c^	FM	T	CHC	MC	TA	pH	*L*	*a*	*b*	EI
	X_1_	X_2_	X_3_	Y_1_	Y_2_	Y_3_	Y_4_	Y_5_	Y_6_	Y_7_
1	19 (0) ^d^	130 (0)	0.13 (0)	13.6	150.7	6.8	22.43	4.01	−0.33	2.13
2	15 (−1.682)	130 (0)	0.13 (0)	10.0	141.1	6.8	28.89	5.01	0.35	3.26
3	19 (0)	130 (0)	0.13 (0)	14.0	154.7	6.8	21.4	3.83	−0.35	2.05
4	19 (0)	130 (0)	0.13 (0)	13.3	130.4	6.8	22.38	4.00	−0.04	2.19
5	21.38 (1)	141.89 (1)	0.2 (1)	12.3	174	6.9	21.47	3.69	−0.38	1.78
6	19 (0)	130 (0)	0.13 (0)	13.2	153.8	6.9	20.96	3.76	−0.12	2.19
7	19 (0)	130 (0)	0.25 (1.682)	13.1	143.5	6.7	22.49	4.17	0.11	2.02
8	21.38 (1)	118.11 (−1)	0.05 (−1)	11.9	165.5	6.8	19.44	4.13	−0.17	1.72
9	19 (0)	130 (0)	0 (−1.682)	13.1	142.6	7.1	23.44	3.73	−0.40	2.33
10	21.38 (1)	141.89 (1)	0.05 (−1)	11.6	176.9	6.7	20.17	4.05	−0.22	1.80
11	16.62 (−1)	141.89 (1)	0.05 (−1)	8.5	170.8	6.7	25.72	4.77	0.32	2.54
12	16.62 (−1)	118.11 (−1)	0.2 (1)	9.4	144.5	6.8	23.96	4.33	0.12	2.62
13	23 (1.682)	130 (0)	0.13 (0)	14.7	149.5	7.0	21.18	3.83	−0.28	1.71
14	21.38 (1)	118.11 (−1)	0.2 (1)	12.4	183.4	6.8	20.83	3.58	−0.32	1.76
15	19 (0)	110 (−1.682)	0.13 (0)	11.0	163.3	6.8	21.21	4.25	−0.23	2.21
16	16.62 (−1)	141.89 (1)	0.2 (1)	8.0	169.1	6.8	29.28	4.97	0.14	2.80
17	19 (0)	150 (1.682)	0.13 (0)	10.1	153	6.8	22.35	4.94	−0.25	2.08
18	16.62 (−1)	118.11 (−1)	0.05 (−1)	9.7	153.8	6.7	24.7	4.2	0.41	2.58
19	19 (0)	130 (0)	0.13 (0)	13.0	142.3	6.9	20.73	3.87	−0.44	2.14
20	19 (0)	130 (0)	0.13 (0)	13.1	157.4	6.9	21.63	3.73	−0.33	2.06

^a^ FM = Feed moisture (%), T = Final extruder temperature (°C), CHC = Calcium hydroxide concentration (%); ^b^ MC = Moisture content (%), TA = Total anthocyanins (mg·kg^−1^), *L* = White (100) to black (0), *a* = Red (+) to green (−), *b* = Yellow (+) to blue (−), EI = Expansion index; ^c^ Tr = Treatment; ^d^ Numbers in parentheses corresponded to coded values.

According to the results presented in Table 2, the extrudates produced at higher FM and T had greater losses of moisture content. The evaporation of water when the product emerged from the die provoked proportional losses of moisture from 11% up to 43% when compared with the original FM. It has been reported that after the extrudate is released from the die and reaches the maximum expansion, the product starts to contract under elastic recoil. The sudden drop of temperature diminishes the viscosity, causing the evaporation of 8–10 g of moisture per 100 g of fluid [12].

### 2.2. Effects of FM, CHC and T on Total Anthocyanins (TA)

The ANOVA (Table 1) showed that the quadratic term (T)^2^ was the processing factor that had the most significant effect (*p* < 0.0459). The model fitting for TA in terms of the actual factors is presented in Equation (2):

Y_TA_= 960.61 − 12.61(T) + 0.049(T)^2^ (2)

**Figure 2:**
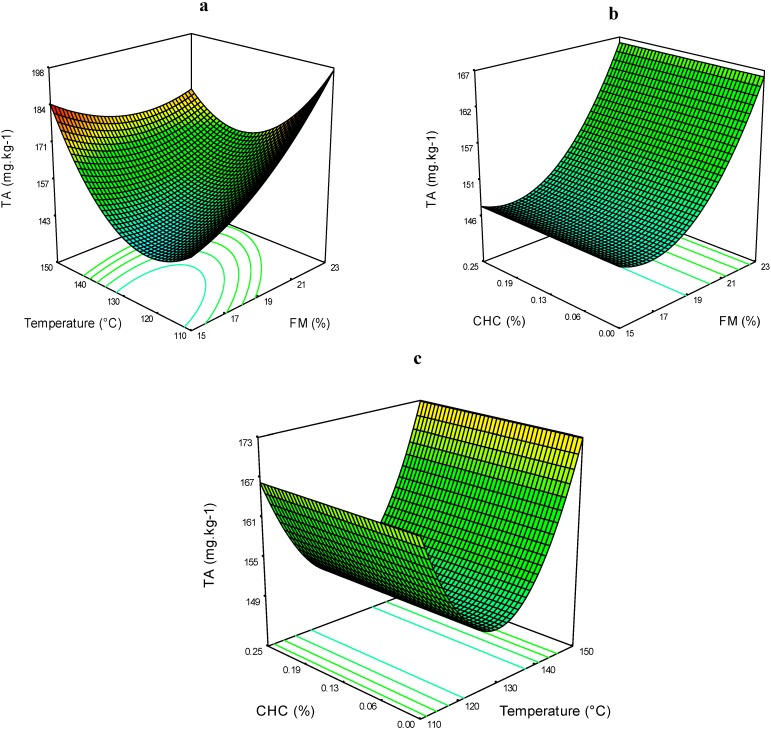
Total anthocyanins (TA) of expanded nixtamalized blue corn extrudates, as a function of: (**a**) feed moisture (FM) and temperature (T); (**b**) feed moisture (FM) and calcium hydroxide concentration (CHC); and (**c**) temperature (T) and calcium hydroxide concentration (CHC).

The values obtained from this analysis ranged between 130.4 and 183.4 mg·kg^−1^ (Table 2). The interaction effects of FM*T on the TA are shown in Figure 2a; at higher levels of these process factors, the TA levels were the highest. At lower FM and T levels, the TA was the lowest. These results are inconsistent with other studies where it was demonstrated that at higher temperatures, the destruction of anthocyanins increased. Temperature has a notable effect on anthocyanin structures; these compounds lose their color at elevated temperatures, and they become paler because the equilibrium among the anthocyanin species shifts towards other chemical forms [4].

However, the results obtained in our research are consistent with those from a previous study [7], where it was demonstrated that even when the total anthocyanin content decreased due to higher processing temperatures, there was an increment of 11.3% in the cyanidin 3-glucoside (major anthocyanin in blue corn) content. Other authors [13] showed that purified anthocyanins degraded at a faster rate than anthocyanins in unpurified extracts obtained from the food material. These findings lead to the presumption that some anatomical parts of the grain (like the pericarp) could be acting as protectors of the anthocyanins, reducing their degradation by high processing temperatures.

In Figure 2b, it can be observed that there was no effect of CHC on AT content; rather FM had a greater effect such that at high levels of this process factor, the TA was slightly higher at any given CHC level. The interaction effect of T*CHC is presented in Figure 2c, where TA contents were higher at temperatures of 141.89 and 118.11 °C.

Nevertheless, it has been reported that many factors affect the stability of these compounds, such as extraction procedures and glycosylated substituents, which could affect the total anthocyanin content in the extrudates samples [14].

### 2.3. Effects of FM, CHC and T on pH

There were no significant effects of the extrusion processing factors on pH values (Table 1). This was probably due to the minimum calcium hydroxide concentrations (range 0%–0.25%) used in the treatments to obtain the extrudates. These results differ from those obtained by Zazueta *et al.* [15], who reported that the CHC had an effect on physical and chemical properties of blue maize extrudates elaborated by extrusion. In the case of the pH, alkaline media induced starch swelling and gelatinization, which expose reactive sites of the starch. There are formation of starch-calcium complexes that changes conformational and structural characteristics of the starch based foods. The pH ranged from 6.7 to 6.1 in all the treatments (Table 2). It can be seen that at the CHC used in this study, the anthocyanin content could not be affected by pH, allowing the manipulation of other extrusion parameters.

### 2.4. Effects of FM, CHC and T on Color Parameters (L, a, b)

**Figure 3:**
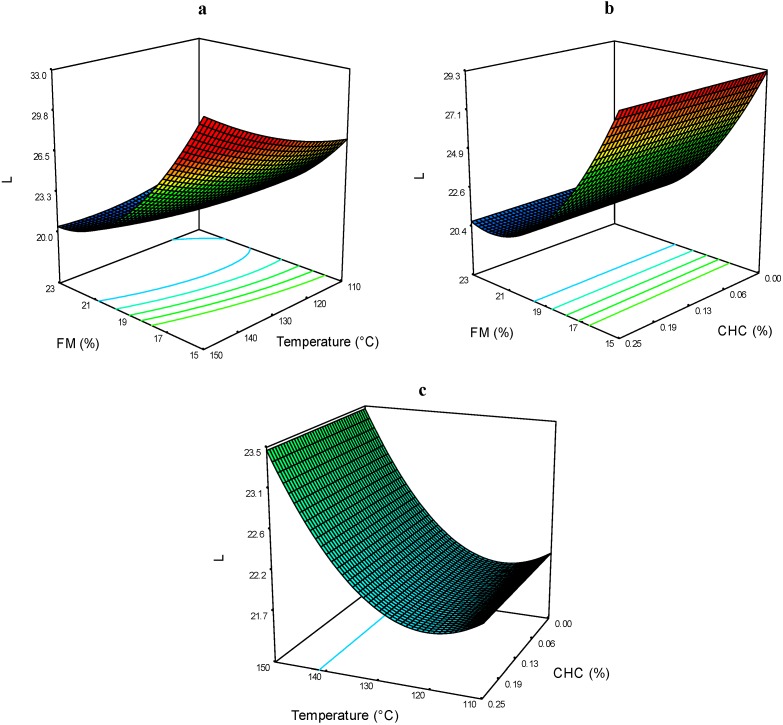
Color parameter *L* of expanded nixtamalized blue corn extrudates, as a function of: (**a**) feed moisture (FM) and temperature (T); (**b**) feed moisture (FM) and calcium hydroxide concentration (CHC); and (**c**) temperature (T) and calcium hydroxide concentration (CHC).

Luminosity (*L*) was evaluated on a scale of 100 (white) to 0 (black). Extrudates with lower values of *L* appeared darker (purple) to the unaided eye. The ANOVA (Table 1) showed that the FM affected the color parameter *L* very significantly in linear (*p* < 0.0001) and quadratic (*p* < 0.0040) terms. The values of this parameter ranged between 19.44 and 28.89 (Table 2). Figure 3a,b show that at low FM levels and high CHC and T, the *L* parameter reached the highest values. The interaction T*CHC had no significant effect on *L* values (Figure 3c).

The model fitting for *L* in terms of the actual factors is presented in Equation (3):

Y*_L_* = 109.27 − 8.10(FM) + 0.185(FM)^2^ (3)

When anthocyanins are exposed to higher temperatures, some degradation reactions occur, forming colorless structures that fade the original red-blue coloration. It has been reported that in corn extrudates the parameter *L* was highly affected by the FM of the extrusion process. It can be seen that at higher processing temperatures, the lightness (*L*) increased in the blue corn extrudates (Figure 3a,c). Some authors [16] have reported an increase in the lightness of anthocyanin extracts containing cyanidin and pelargonidin with mono- and diglucoside moieties. This effect was attributed to the transition of the colored flavylium cation into colorless and yellowish carbinol and chalcone forms, respectively. It is possible that the same anthocyanin degradation mechanism occurred in the extrudate samples obtained in this study.

The positive and negative values of the color parameter *a* indicate red and green shades, respectively. The ANOVA (Table 1) showed that the linear terms of FM (*p* < 0.0001) and T (*p* < 0.0012) presented a highly significant effect. The quadratic terms of these processing variables also had very significant effects: FM^2^ (*p* < 0.0013) and T^2^ (*p* < 0.0001). The interactions FM*T and FM*CHC showed significant effects (*p* < 0.0191 and *p* < 0.0140, respectively).

The model fitting of *a* in terms of the actual factors is presented in Equation (4):

Y*a* = 27.92 − 0.48(FM) − 0.30(T) + 16.96(CHC) − 0.0052(FM)(T) − 0.88(FM)(CHC) + 0.029(FM)^2^ + 0.0016(T)^2^ (4)

**Figure 4:**
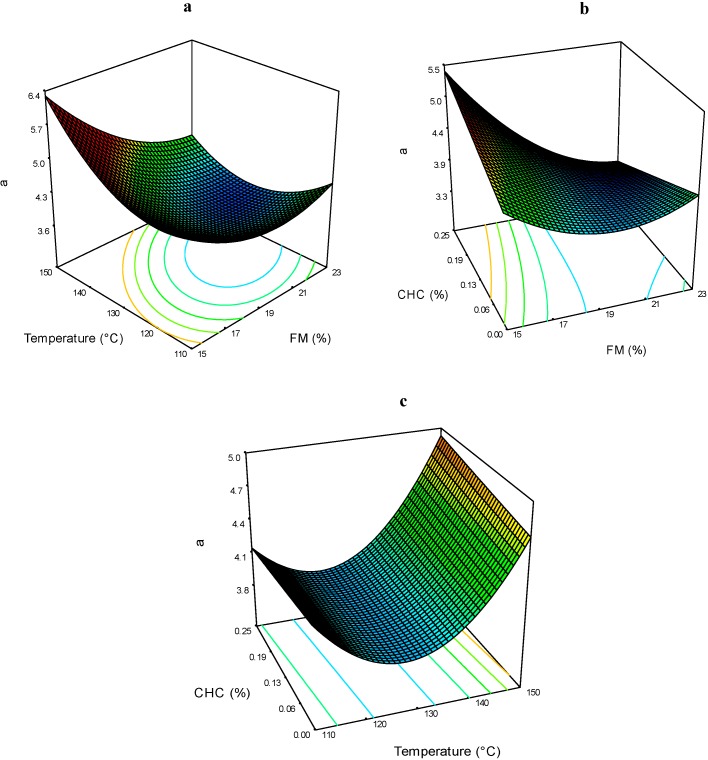
Color parameter *a* of expanded nixtamalized blue corn extrudates, as a function of: (**a**) feed moisture (FM) and temperature (T); (**b**) feed moisture (FM) and calcium hydroxide concentration (CHC); and (**c**) temperature (T) and calcium hydroxide concentration (CHC).

The color parameter *a* of the extrudates ranged between 3.58 and 5.01 (Table 2). The effects of the extrusion processing conditions are presented in Figure 4a–c. It can be seen that the three processing factors affected this evaluation. The FM*T interaction shows that at low levels of FM and high T, the color parameter *a*, is the highest (Figure 4a). In Figure 4b, the FM*CHC interaction, shows that as the FM diminishes and CHC increases, the values for color parameter *a* are higher. The stability of anthocyanins is highly dependent on pH at levels of 6–7 or higher. The coloration of these compounds changes to blue-purplish and the quinoidal forms are degraded rapidly by the air oxidation, thereby decreasing the positive *a* values.

Figure 4c shows the effects of T*CHC on the color parameter *a*, and it can be seen that at higher T, the *a* values increases by increasing CHC. It is probably that the release of acylated anthocyanins increases the relative proportion of the stable red flavylium cation, thus protecting the red coloration at higher pH [3]. The effect of CHC in the color parameter *a* of pigmented nixtamalized products was evaluated by other authors [17], concluding that extrusion process improves the retention of anthocyanins and produced more retention of blue-red colorations in the obtained products.

In the color spectrum, positive and negative *b* values indicate yellow and blue shades, respectively. The ANOVA (Table 1) results showed that the FM had a highly significant effect on this parameter in its linear term (*p* < 0.0008) and a significant in its quadratic term (*p* < 0.0325).

The model fitting of *b* in terms of the actual factors is presented in Equation (5):

Y*_b_*= 9.16 − 0.89(FM) + 0.0208(FM)^2^ (5)

**Figure 5:**
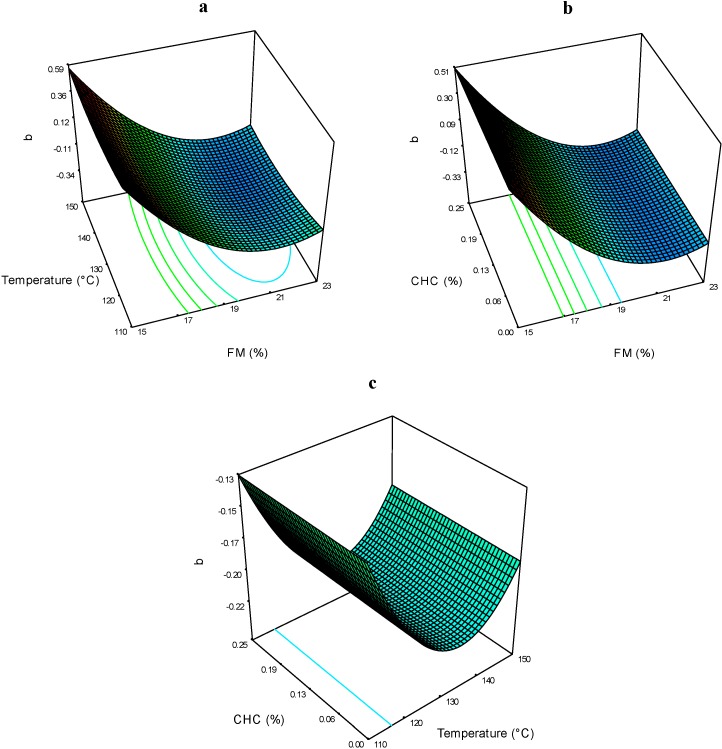
Color parameter *b* of expanded nixtamalized blue corn extrudates, as a function of: (**a**) feed moisture (FM) and temperature (T); (**b**) feed moisture (FM) and calcium hydroxide concentration (CHC); and (**c**) temperature (T) and calcium hydroxide concentration (CHC).

The values of *b* obtained in the extrudates ranged from −0.44 to 0.41 (Table 2). Figure 5a shows the effects of FM*T on the color parameter *b*. The FM had a significant effect: as the FM increased, the *b* values became positive, and practically no effects of T. The effect of FM*CHC on color parameter *b* is presented in Figure 5b, which shows the same trend as that in the FM*T interaction. As the FM decreased, the parameter *b* increased regarding the CHC. Due to that anthocyanins are pH-dependent compounds, changes in this parameter were expected. However, the presence of one or more acyl groups in the molecule prevents the hydrolysis of the flavylium form and promotes the synthesis of quinoidal structures with blue shades, making these types of compounds more stable and less sensitive to pH changes [4]. The effects of T*CHC on color parameter *b*, are presented in Figure 5c. It can be observed that *b* remained constant (non-significant effect) regarding the CHC and T. It can be assumed that the acylated anthocyanins contained in the blue corn extrudates, were not affected by these processing factors.

### 2.5. Effects of FM, CHC and T on Expansion Index (EI)

The ANOVA (Table 1) showed that the linear and quadratic terms of the FM had a very significant effect in the EI values (*p* < 0.0001 and *p* < 0.0037, respectively).

The model fitting of IE in terms of the actual factors is presented in Equation (6):

Y*_EI_* = 12.75 – 0.931(FM) + 0.019(FM)^2^ (6)

**Figure 6:**
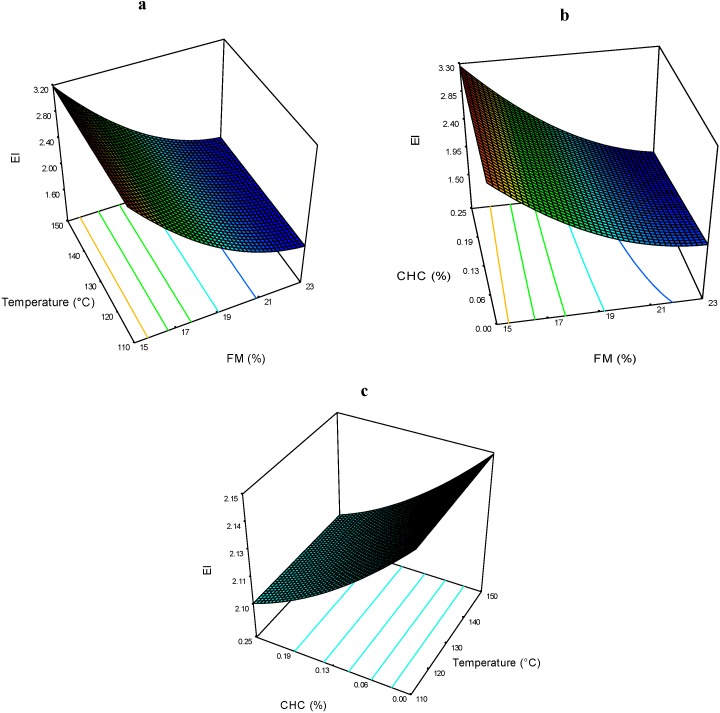
Expansion index (EI) of expanded nixtamalized blue corn extrudates, as a function of: (**a**) feed moisture (FM) and temperature (T); (**b**) feed moisture (FM) and calcium hydroxide concentration (CHC); and (**c**) temperature (T) and calcium hydroxide concentration (CHC).

The EI of the extrudates ranged from 1.71 to 3.26 (Table 2). The effects of FM*T on the EI are presented in Figure 6a. At any T, the FM had a significant effect, demonstrating that the higher the FM, the lower the EI. The same trend was observed for FM in the FM*CHC interaction (Figure 6b), where CHC had no significant effect on the EI; in addition the T*CHC interaction had no effect on the EI (Figure 6c).

The effects of FM on the extrudate expansion have been widely studied and its role affecting this parameter was established because of the sudden drop of pressure (from the inside of the extruder barrel to the atmospheric pressure). The pressure drop causes an extensive expulsion of water vapor from the melt, which emerges in the form of bubbles and allows the expansion of the molten extrudate [18]. The results obtained in our study were in according with those obtained by Thymi *et al.* [19], who reported that the extrudate expansion is most dependent on the material moisture content, as higher FM decreased the expansion of corn extrudates. The effects of temperature on the expansion of starch-based extrudates have also been reported; however, in the expanded extrudates, the effects of this processing factor were not statistically significant, possibly because the temperature range (110–150 °C) was not sufficiently large.

### 2.6. Optimization and Model Prediction Performance

In this part of the study, optimization was defined as the processing conditions that provided an optimum value as a function (maximum or minimum) of certain variables subject to constraints that were previously imposed [20]. Cereal snack products containing natural compounds with additional health benefits (such as antioxidants) are highly accepted by consumers. In addition, texture and appearance (attractive color) are two of the most important sensorial characteristics for these products.

The goals for optimization in this study were to maximize the total anthocyanin content and the expansion index in order to obtain extrudates with the highest amount of antioxidants (anthocyanins) and acceptable crunchiness, assuming that the higher the expansion index, the crunchier the extrudate. In the case of color parameter *b*, the goal was to minimize the value so that the blue/purplish coloration was more intense, making the appearance of the extrudates more attractive.

**Figure 7:**
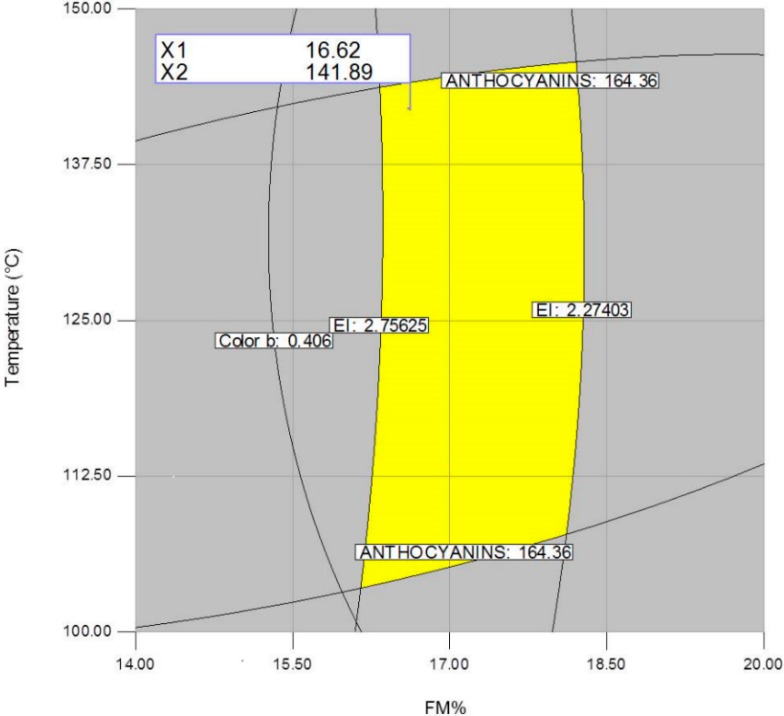
Overlay plot and optimized region (best combination) of the extrusion processing factors feed moisture (FM) and temperature (T), for producing expanded nixtamalized blue corn extrudates.

**Figure 8:**
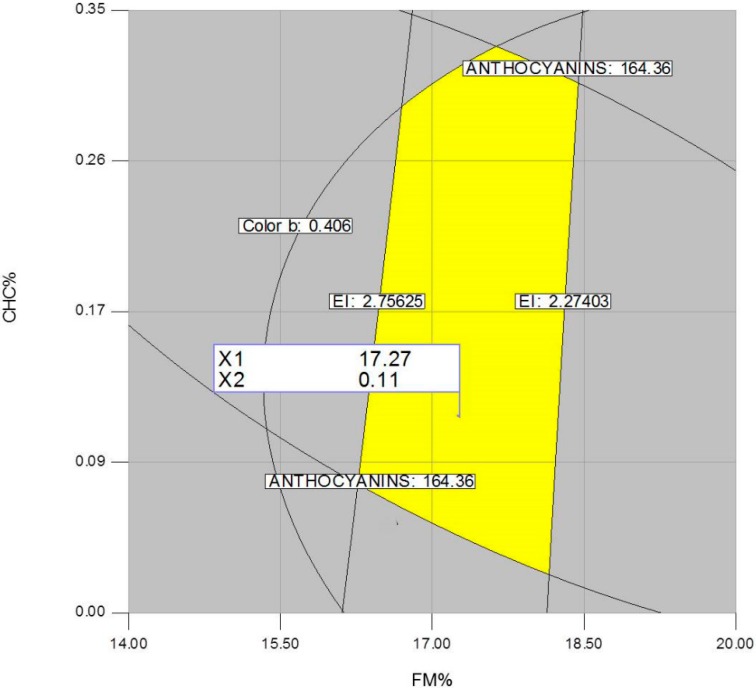
Overlay plot and optimized region (best combination) of the extrusion processing factors feed moisture (FM) and calcium hydroxide concentration (CHC), for producing expanded nixtamalized blue corn extrudates.

**Figure 9:**
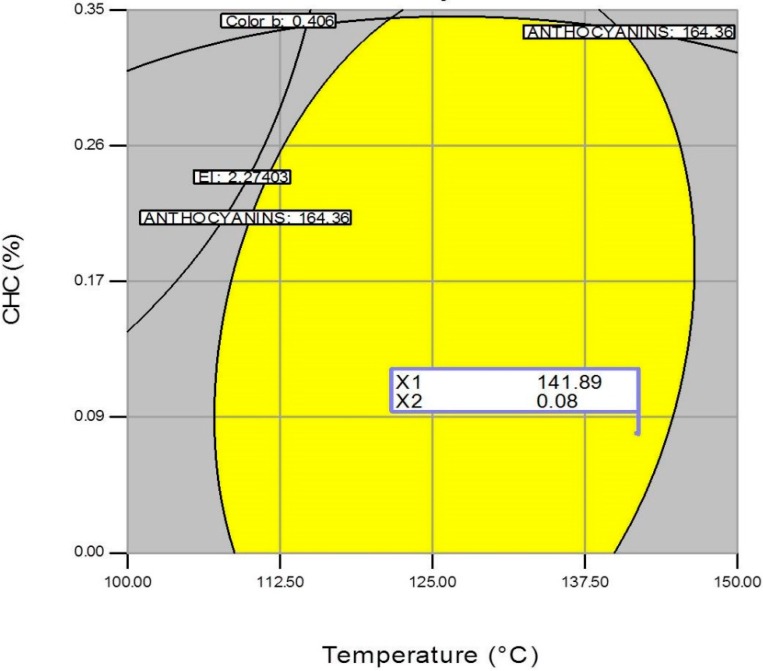
Overlay plot and optimized region (best combination) of the extrusion processing factors temperature (T) and calcium hydroxide concentration (CHC), for producing expanded nixtamalized blue corn extrudates.

Figure 7, Figure 8 and Figure 9 show the central points of the best combination regions (optimum) corresponding to the following processing factors: FM(%) = 16.62/T(°C) = 141.89, FM(%) = 17.27/CHC(%) = 0.11, and T(°C) = 141.89/CHC(%) = 0.08. From these three set of values, an average one was computed for each processing factor, resulting in the following processing factors: FM(%) = 16.94, T(°C) = 141.89 (fourth zone of the extruder) and CHC(%) = 0.095. These conditions estimated the production of expanded nixtamalized blue corn extrudates with a total anthocyanin content of 160 mg·kg^−1^, expansion index of 2.66, and color parameter *b* of 0.10.

#### Evaluations of the Expanded Nixtamalized Blue Corn Snacks Produced with the Optimum Extrusion Conditions

The experimental validation of the processing factors was performed in the extruder with the conditions obtained by the overlay plots. Results of the chemical and physical characteristics evaluated in the nixtamalized blue corn expanded extrudates are presented in Table 3.

**Table 3:** Predicted values and experimental results of chemical and physical evaluations on the expanded nixtamalized blue corn extrudates obtained from the optimum processing conditions.

Response Variable	Predicted Value	Experimental Value
TA (mg·kg^−1^) ^a^	160	158.87 ± 2.26 ^c^
Color *b* ^b^	0.10	−0.45 ± 0.08 ^c^
Expansion Index	2.66	3.19 ± 0.11 ^d^

^a^ Total anthocyanins; ^b^ *b* = Yellow (+) to blue (−); ^c^ Average ± standard deviation, *n* = 4; ^d^ Average ± standard deviation, *n* = 40.

Despite the use of biological material (whole blue corn), the model’s prediction led to highly accurate results. The experimental value for TA in the extrudates was 158.87 mg·kg^−1^ (estimated, 160 mg·kg^−1^), meaning 99.2% fitting. The experimental result for the expansion index was 3.19. This parameter was higher than expected (estimated, 2.66), representing 83.3% fitting and more expansion (crunchiness) in the extrudate. Finally, the experimental result for the color parameter *b* was −0.45. This result was better than expected (estimated, 0.10), meaning that the negative values indicated an increased intensity of the blue/purple shades.

## 3. Experimental Section

### 3.1. Raw Material

Creole soft blue corn was obtained in Toluca, Mexico (2010 crop). The grains (10 kg) were cleaned (Clipper BLOUNT/Ferrell-Ross, Model M2BC; Blufton Inc., Blufton, IN, USA) and ground in a six blade mill with rubbed shell (Pulvex SA de CV, Model 200, serial 1030401, Mexico, DF, Mexico) and passed through a 0.8 mm mesh. The ground corn was stored in sealed polyethylene bags and kept in the dark (to avoid anthocyanin degradation) at 5 °C until use. Commercial lime (calcium hydroxide) (Calhidra de Sonora, SA de CV, Hermosillo, Son., Mexico) and distilled water were used.

### 3.2. Extrusion Process

Samples (300 g each) of ground corn were mixed in a laboratory blender (Kitchen Aid, Model MK45SSWH, St. Joseph, MI, USA), with different concentrations of calcium hydroxide (0%–0.25%) and distilled water (15%–23%) for 5 min. Each ground corn sample was conditioned with calcium hydroxide and water according to the experiment design (Table 2). The conditioned ground corn samples were kept in sealed polyethylene bags at 5 °C in dark conditions for 12 h before extrusion.

A single-screw extruder (Brabender Instruments, Model E19/25 D, OHG, Duisburg, Germany) with four heat/cool zones was used. The temperatures inside the barrel in the first, second and third zones were 60, 80, 110 °C, respectively, and the fourth zone temperature was set according to the experiment design (110–141.89 °C). The conditioned ground corn was fed into the extruder under the following conditions: screw number 3 (nominal compression ratio 3:1 and diameter 19 mm); screw speed of 120 rpm; hopper feed rate of 50 rpm; and 3 mm die opening diameter. The obtained extrudates were cooled at room temperature (25 °C), dried at 60 °C for 30 min in a tunnel dryer, and then stored at 5 °C in sealed polyethylene bags in the dark until analysis.

### 3.3. Extrudate Evaluations

#### 3.3.1. Moisture Content (MC)

The AACCI Approved Method 44–15.02 [21] was used, and three replicates of the analysis were performed for each treatment

#### 3.3.2. Total Anthocyanins (TA)

The analysis was assessed according to Abdel-Aal and Hucl [22]. Samples (3 g) of ground extrudates were weighed in a 50 mL centrifuge tube, and acidified ethanol (ethanol with 1 N HCl, 85:15 v/v, 24 mL) was added. The solutions were adjusted at pH 1 with 4N HCl and then shaken and centrifuged (Thermo Scientific, Model Heraes Biofuge Primo R., Dreieich, Germany) at 27,200× *g* for 15 min; this step was performed twice. The supernatant was separated into a centrifuge tube, and the volume was adjusted to 50 mL with acidified ethanol. The absorbance was measured at 535 nm (against a blank) in a UV-visible spectrophotometer (Varian Australia PT LTD, Cary 50 CONC, Victoria, Australia). The analysis was made in triplicate.

#### 3.3.3. pH

This determination was measured according the AACCI Approved Method 02–52 [23]. Three replicates were performed.

#### 3.3.4. Color

The parameters *L*, *a*, and *b* for each treatment were measured using a Hunter Lab Miniscan XE Plus (Hunter Association Laboratories, Reston, VA, USA). The color value *L* indicates lightness on a scale of 100 (white) to black (0), positive and negative *a* color values indicate red and green shades, respectively, and positive and negative *b* color values indicated yellow and blue shades, respectively. These measurements were made in triplicate for each treatment.

#### 3.3.5. Expansion Index (EI)

The expansion index was measured using a Digital Caliper (Mitutoyo Corp., Model CD–6 CS, Kanagawa, Japan) and was calculated as the ratio of the extrudate diameter to the diameter of the extruder die (3 mm). Forty replicates of each determination were performed.

### 3.4. Experimental Design and Statistical Analysis

**Figure 10:**
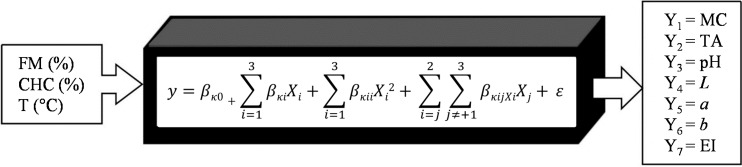
Empirical model of the interaction between processing and response variables.

A central composite experimental design of three factors and five levels was used (Table 2). The independent variables were: feed moisture, FM (X_1_, 15%–23%); calcium hydroxide concentration, CHC (X_2_, 0%–0.25%) and fourth zone extruder temperature, T (X_3_, 110–150 °C), coded with levels of −1.682, −1, 0, +1 and +1.682. The response variables in the extrudates were: moisture content (%), total anthocyanins (mg·kg^−1^), pH, color parameters (*L*, *a*, *b*) and expansion index (EI). The empirical model representing the interaction between the independent and response variables is presented in Figure 10.

This mathematical expression is used to model the response variables (Y_1_–Y_7_) where the *k* value changes from 1 to 7; where β*_k0_* represents a constant, β*_ki_* the linear coefficient, β*_kii_* the quadratic coefficient, β*_kji_* the interaction effect of the response variables, and ɛ the experimental error.

All data obtained from the response variables were recorded, and an analysis of variance (ANOVA) was performed with a confidence level of 95%. Besides, a backward regression analysis was applied, and non-significant factors (*p* > 0.1) were eliminated from the second-order polynomial equation. Then a new equation was recalculated to achieve the final predictive model for each response variable. Response surface methodology (RSM) was used [24]. Contour plots for each determination were obtained using Design Expert Software V.7.0.0 (Stat-Ease, Minneapolis, MN, USA).

### 3.5. Optimization of the Extrusion Process

To obtain the best combinations of factors for the extrusion process (FM, CHC, T), response surface methodology was used. The response variables to optimize the process were: total anthocyanins (maximize), color *b* (minimize) and expansion index (maximize). Once the graphical contour plots were obtained, superposition surface methodology was applied to achieve the optimization technique [17]. Three contour plots were made, and the optimum combination of the processing variables were selected. Validation of the optimization model was performed according to the estimated processing values.

## 4. Conclusions

The FM, in linear and quadratic terms, was the factor that most significantly affected all of the evaluations performed in the extrudates, except for the TA determinations, where the quadratic term of T showed the most significant effect. The color parameter *a* was the response variable that was most significantly affected by the three processing factors and their interactions. Certainly during the process, there are complex transformations in anthocyanins showed by the effect of the extrusion factors in the color parameter *a*. The TA content was not affected either by FM or CHC, leaving the processing temperature as the most important factor influencing the retention of anthocyanins during the elaboration of this kind of products.

According to the overlay plots, the optimum extrusion conditions to obtain expanded nixtamalized blue corn extrudates were: FM(%) = 16.94, CHC(%) = 0.095 and T(°C) = 141.89, and the estimated values of TA, EI and color parameter *b* were: 160 mg·kg^−1^, 2.66 and 0.10, respectively. The expanded nixtamalized blue corn extrudates obtained experimentally under those conditions were: TA 158.87 mg·kg^−1^; EI 3.19; and color parameter *b*, −0.44 (intense blue/purple coloration).

The surface response methodology was a useful tool to obtain an expanded nixtamalized blue corn snack with anthocyanins and acceptable texture and color. Extrusion process and the anthocyanin content of expanded nixtamalized snacks can be optimized to obtain functional products.
